# Insaka: mobile phone support groups for adolescent pregnant women living with HIV

**DOI:** 10.1186/s12884-021-04140-6

**Published:** 2021-09-30

**Authors:** Nikita Simpson, Anna Kydd, Mwelwa Phiri, Madalitso Mbewe, Lucheka Sigande, Thomas Gachie, Malebo Ngobeni, Tebogo Monese, Zuzana Figerova, Hugo Schlesinger, Virginia Bond, Steve Belemu, Musonda Simwinga, Ab Schaap, Maurice Biriotti, Sarah Fidler, Helen Ayles

**Affiliations:** 1 Department of Anthropology, London School of Economics and Political Science, London, UK; 2 SHM Foundation, 20-22 Bedford Row, London, UK; 3 Department of Infectious Diseases, Imperial College, London, UK; 4 Zambart, Lusaka, Zambia; 5 Department of Global Health and Development, London School of Hygiene and Tropical Medicine, London, UK; 6 Clinical Research Department, London School of Hygiene and Tropical Medicine, London, UK

**Keywords:** Mobile phones, Pregnancy, Adolescents, Psychosocial support, Peer support

## Abstract

**Background:**

Mobile phone-based interventions have been demonstrated in different settings to overcome barriers to accessing critical psychosocial support. In this study, we aimed to assess the acceptability and feasibility of a phone-based, peer-to-peer support group intervention for adolescent pregnant women aged 15–24 years living with HIV in Zambia.

**Methods:**

Sixty-one consenting participants were recruited from Antenatal Clinics of two large urban communities in Lusaka. They were invited to participate in the mobile phone-based intervention that allowed them to anonymously communicate in a small group led by a facilitator for 4 months. A mixed methods approach was used to assess acceptability and feasibility, including a focus group discussion, pre- and post-intervention interview and analysis of the content of the text message data generated.

**Results:**

Participants reported finding the platform “not hard to use” and enjoyed the anonymity of the groups. Seventy-one percent of participants (*n* = 43) participated in the groups, meaning they sent text messages to their groups. Approximately 12,000 text messages were sent by participants (an average of 169 messages/user and 6 mentors in 6 groups. Topics discussed were related to social support and relationships, stigma, HIV knowledge and medication adherence.

**Conclusion:**

The study showed that the intervention was acceptable and feasible, and highlighted the potential of the model for overcoming existing barriers to provision of psychosocial support to this population.

**Supplementary Information:**

The online version contains supplementary material available at 10.1186/s12884-021-04140-6.

## Background

Routine HIV testing in antenatal settings means that pregnancy is frequently the point at which young women first discover their HIV-status [1]. While this has significantly improved Anti-Retroviral Treatment (ART) coverage amongst this group in Southern Africa - with 92% of pregnant women receiving ART in Zambia in 2017 [2, 3] - adolescent pregnant women, are known to have poorer access to and engagement with HIV services [4]. Women who are diagnosed with HIV either immediately before, or during pregnancy are more likely to be non-adherent to ART [5]. Experiencing stigma during pregnancy, compounded with the difficulties of managing a chronic condition like HIV alongside motherhood, can have negative health consequences for mothers [6] and negative developmental consequences for their baby [7]. Further, concerns about HIV status and testing have been shown to increase mental distress in pregnant women [8].

In Zambia, HIV prevalence among young women is more than double that of young men (5.7% of young women were living with HIV in 2017, compared to 2.5% of young men) [9]. As adolescent girls are among those at highest risk of HIV acquisition [10], and pregnancy rates in this age group remain high [11], they are likely to represent both a significant proportion of all pregnant women infected with HIV, and perhaps most likely to be neglected by existing research and interventions [12].

Improving social support has been shown to have a positive impact on adherence to medication across a range of chronic illnesses for vulnerable populations [13]. There is evidence to suggest that support groups and peer mentorship are effective interventions for delivering psychosocial support to pregnant women living with HIV [14, 15]. In particular, where there are significant barriers to attending face-to-face counselling or support groups including time limitations - due to employment or caring responsibilities in the household, a lack of space in health facilities, a lack of funds for transport, the coercive control of male partners, stigma preventing mobility or social distancing regulations [16] - technological interventions have an important place. Indeed, mobile phone-based interventions can overcome barriers that people have to accessing critical social and psychosocial support [17]. Recent studies in sub-Saharan Africa have moved beyond the use of the mobile phone as an educative tool, and toward its potential as a device for behaviour change and psychosocial support for populations living with HIV [18, 19].

The mobile phone-based, virtual peer support group intervention used in this study was developed in 2008 by the SHM Foundation in Jalisco, Mexico to respond to the negative mental health impact of social isolation and stigma that many of those living with HIV were experiencing. The SHM Foundation have piloted and evaluated this model in a number of countries with a range of populations, health conditions and age groups [20]. In South Africa, the delivery of psychosocial support to HIV positive pregnant women via mobile phone support groups to prevent mother to child transmission was proven to be acceptable and feasible [21]. In Guatemala, the intervention was successful at providing psychosocial support to new mothers [22]. The aim of this study, Project Insaka, was to assess the feasibility and acceptability of a mobile phone-based support group intervention for HIV positive pregnant women aged 15–24 in Lusaka, Zambia.

## Methods

### Study setting

The Insaka pilot study was a process evaluation conducted in two low-income, high-density urban communities in Lusaka, Zambia. According to the PopART census of 2013 (formative research in a universal test and treat study in Zambia and South Africa), the average size of each population was 100 200 [23]. As of 2013, slightly over 50% of the populations were aged 18 years and older. Although Nyanja and Bemba are the most commonly spoken languages in both communities, which are comprised of multilingual ethnic groups and a mix of Zimbabwean, Rwandese and Congolese foreign nationals [24]. The healthcare needs in each community are serviced by one government clinic, which provides both in- and out-patient services. The health facilities were initially designed (and built in the post-colonial period) for smaller populations, but due to rural-urban migrations and the formation of informal settlements, the populations and catchment areas served by the local government health facilities have grown. The average HIV prevalence of the two communities is 12%.

### Participants

Sixty-one participants were recruited from hospital Antenatal Clinics of two large peri-urban communities in Lusaka, Zambia. The participants were purposively recruited from the health facilities’ Mother and Child (MCH) department by Peer Mentors and District Project coordinators based at the facility under Zambart. Participants were eligible for the study if they were between 28 and 34 weeks pregnant at the time of recruitment, aged between 15 and 24 years and were confirmed to be living with HIV. Participants were explained the parameters of the study and asked to give informed consent to participate. If they were below the age of 18, they had to have disclosed their HIV status to guardians or parents as they were required to have parental/guardian consent to participate in the study.

The sample size of this study was determined by the resources available to the study, the limited time frame for recruitment, and the capacity of the research team to monitor the support group conversations. It was not powered to produce statistically significant results.

### Procedures

The research team conducted a participatory design workshop with a group of pregnant women living with HIV and key stakeholders in Lusaka to gather their stressors, psychosocial support needs and mobile phone use behaviours. Participants discussed their social support needs, barriers to accessing services and technology use behaviours. This information was used to adapt the mobile phone support group model to the needs of this population. Key adaptations of the model from previous iterations included the transition from an SMS to a digital messaging platform, the use of a smart phone, the inclusion of a core curriculum of topics focused on maternal and child health, intimate partner violence and pregnancy, the inclusion of trained peer support workers (who were also young women) as facilitators and the focus of recruitment in antenatal clinics.

During a launch event, participants were given a mobile smart phone device (ITEL 1503 mobile devices, running the Android 4.4 KitKat® operating system, and kiosked (designed the interface so they did not have other apps on them), with pre-loaded mobile data. They were asked to choose a nickname and remain anonymous for the duration of the intervention. They were encouraged to keep the content confidential and not to share their phone. Participants were placed in six virtual groups of 6–8 participants for a period of 4 months between July and November 2018, over the peri-partum and post-partum period. The support groups used the open-source technology, Rocket.Chat®, which we adapted to meet our needs. This platform ensured participants could not share numbers, forward messages, take or send photo or video content. The research team could administer the groups and monitor conversations. The plug-in Zapier® allowed messages to be saved securely for future analysis. During the running of the groups, the participants could freely communicate amongst themselves anonymously. Groups were facilitated by a trained Peer Mentor (PM) who delivered a curriculum of topics developed in consultation with the project team and key stakeholders. There were a number of sessions where health professionals – a gynaecologist, a nutritionist and a general practitioner - were invited into the groups to run a session on a particular topic, where participants could ask specific medical questions, as informed by information gathered in the participatory workshops at the beginning of the study. At the end of the groups, participants were asked to keep their phones, but the messaging platform was deleted from their phones in order to prevent ethical and information accuracy concerns surrounding conversations that were unmonitored or unfacilitated. They were invited to a closing event where the focus group discussion and post-intervention interviews were conducted.

### Data collection

A mixed methods approach was used to assess acceptability and feasibility of the intervention.

#### Pre and post intervention interviews

All participants engaged in a pre- and post-intervention semi-structured interview, delivered by the research team and peer mentors, at the clinic facility. This data was collected using a questionnaire on an electronic tablet using Open Data Kit (ODK)® - a free and open-source tool used to manage mobile data collection. The data collected was later synchronised to the Google Cloud® Server.

The questionnaire was adapted for the Zambian context from an existing iteration of the model run with HIV positive adolescents in Pretoria, South Africa (see Additional File 1). The questionnaire collected demographic data on the age, education level, marital status, employment status and prior pregnancies of participants. The questionnaire included a measure of Stigma measured using the Berger stigma scale [25] and its adapted version in the Serithi Stigma Scale [26]; Perceived Social Support measured using the Multidimensional Measure of Perceived Social Support (MMPSS) [27]; Self Esteem measured using the Rosenberg Self Esteem Scale; HIV Knowledge and Self-Reported Adherence.

#### Focus group discussion

A focus group discussion was conducted by the research team during the closing event with 17 participants (see Additional File 2). No participants who had dropped out of the study were included. Some participants were still pregnant. Participants were asked to reflect on the benefits of using the model and the challenges they faced in using the model. This discussion was transcribed in note form by the research team.

#### Text message data

The research team was able to collect core analytics data from the technology platform. This included the number of messages sent by participants and favoured times of engagement. Through the use of the Zapier® plug-in, anonymised and time-stamped text message data was also collected from the platform and exported to Microsoft Excel® spreadsheets.

### Data analysis

#### Text message content analysis

We assessed the social and psychological content of the messages by conducting a qualitative, thematic analysis of the messages following Hargreaves et al. [28]. 20% of data was purposively sampled (1 in 5 days, from each of the six groups, *n* = 2147 messages). In order to extract this sample, we selected the first of every in 5 days over the 4-month life cycle of the project to provide suitable diversity. Investigators were each given Microsoft Excel® files with the extracted data from one of the six groups. Following the method defined by Braun and Clarke [29], investigators coded and analysed the data to draw out themes.

#### Focus group discussion

Key quotes from the focus group discussion transcript were isolated by the research team.

#### Quantitative analysis

Frequencies and percentages were used to summarise the baseline characteristics of the participants involved in the intervention. Questionnaires were used to conduct pre- and post- intervention interviews, with 5 different specific tools used to investigate HIV knowledge, internalized stigma, anticipated and perceived stigma, social support and low self-esteem.

1. A list of 13 questions with no (scored as 0) and yes (scored as 1) as the possible responses, was used to investigate HIV knowledge.
2. A list of 12 statements with disagree (scored as 0) and agree (scored as 1) as the possible responses, was used to investigate internalized stigma.
3. The “*Berger stigma scale”*, which is a list of 10 Likert items with strongly disagree (scored as 1), disagree (scored as 2), agree (scored as 3) and strongly agree (scored as 4) as the possible responses, was used to investigate anticipated and perceived stigma. A cut-off of > 2 was used and responses > 2 (i.e. agree and strongly agree) scored as 1.
4. The “*multidimensional measure of perceived social support”*, which is a list of 12 Likert items with strongly disagree (scored as 1), disagree (scored as 2), neutral (scored as 3), agree (scored as 4) and strongly agree (scored as 5) as the responses, was used to investigate social support. A cut-off of > 3 was used and responses > 3 (i.e. agree and strongly agree) were scored as 1.
5. The *“Rosenberg self-esteem scale”,* which is a list of 10 Likert items with strongly disagree (scored as 1), disagree (scored as 2), agree (scored as 3) and strongly agree (scored as 4) as the possible responses, was used to investigate self-esteem. Some items of these tool were reverse items and therefore reordered. A cut-off of > 2 was used and responses > 2 (i.e. agree and strongly agree) were scored as 1.

Percentages and graphs were used to summarise the patterns of the way the participants answered the questions in each tool. A mean score outcome was defined for each of the 5 tools by summing up the scores and dividing by the items in each tool. Percentages were used to report the pre- and post-intervention scores. A paired sample t-test was used to compare the differences in mean scores pre- and post-intervention. All analysis was done in Stata® version 15 while graphs were generated in R Version 4.0.0.

## Results

### Participant characteristics

Sixty-one participants were recruited to the study. 29% of the recruited participants (18/61) did not show up for the launch after several attempts of follow-up and were therefore terminated. From Table 1, at baseline, the median age of the active participants was 22 years, with a majority self-reporting to be currently married 74.4% (32/43). 79.1% (34/43) of the active participants self-reported that secondary education was the highest educational level attained, with only 7% (3/43) self-reporting to be currently employed. A majority [74.4% (32/43)] of the participants self-reported that they were pregnant on at least one previous occasion.

**Table 1:** Baseline characteristics of study participants

***Variable***	***%(n/N***^**a**^***)***
*Age in years: Median (IQR*^b^*)*	22 (21–24)
***Marital status***	
*Married or living as married*	97.7.4 (42/43)
*Single*	2.3 (1/43)
***Highest educational attainment***	
*Primary education*	2.3 (1/43)
*Secondary education*	79.1 (34/43)
*Tertiary education*	18.6 (8/43)
*Currently employed: Yes*	7 (3/43)
*Pregnant before*	74.4 (32/43)

^a^*n/N* Number in the level of each factor/active participants

^b^*IQR* Interquartile range

### Uptake and engagement

Of the 61 recruited, 71% of those recruited participated in the groups, meaning they sent text messages (*n* = 43) to their groups. Approximately 12,000 text messages were sent by participants and 6 peer mentors in 6 groups. The three guest speaker sessions were the points of highest engagement over the period and gave the opportunity for participants to ask specific questions. The peak hours of communication – when the majority of messages were sent – were between 18:00 and 21:00. Although there was no particular day that proved to be the *most* popular day to communicate, Sunday was the *least* popular day across all 6 groups. Participants contributed 66% of all messages sent in the groups; peer mentors contributed 27% of messages*;* Admin contributed 7% of messages. Participants sent an average of 169 messages/user; with a daily average of 18 messages/group. Levels of engagement were higher in the first and last months of the intervention. However, there was significant disparity across groups – with groups two (*n* = 440), four (*n* = 559) and five (*n* = 358) reporting higher levels of engagement than others.

In the focus group discussion, participants reported finding the platform “not hard to use”; stating “…It was easy to express oneself [because of anonymity]” and they “…felt cared for in the group”. Indeed, anonymity was a key aspect – “it was good because it was anonymised so no one was able to know who we are.” They enjoyed the fact that there was “no profile photos, no phone numbers or real names showing.” Participants also stated that they enjoyed the groups because they were free to ask questions and “learned about how to look after their baby and their husband.” They enjoyed the sessions with guest medical professionals and creating friendships amongst themselves. Participants also enjoyed the small group setting as they “created friendships amongst [themselves] where [they] would greet each other.” They also enjoyed the face-to-face launch as they liked receiving the “free phones and the food when we met together”.

Participants were frustrated when “the phones would delay to come back when they go for repair”. In the focus group discussion, participants indicated that they disliked how “some people never used to contribute”. They indicated that “sometimes you would post something on the group and people wouldn’t respond so it is like talking to yourself. They would just continue talking about something else and not be interested in what you said.” They expressed displeasure at the end of the intervention that Rocket.Chat® had been deleted from their phones and they could not keep chatting.

Twenty-nine percent of participants (*n* = 18) did not participate in the groups. There are no significant demographic differences between this group and those who participated. In terms of educational attainment, 14 out of the total had attained secondary level, all fall in the 18–24 age group and 13 reported that they were married. Reasons for participants not returning to the groups after recruitment included tensions with their partners about taking part in the project, poor literacy levels and technological issues. However, participants also mentioned that they were able to improve their literacy and technological literacy through the intervention. One participant indicated in the focus group discussion, for instance, “I never knew how to handle a touch screen, but through Insaka I have learnt how to use one.” Another suggested that “I have learnt how to write a message in English and how to spell English words especially because of the spell-check and the speech-to-text option.”

Very quickly over the course of the groups, rules of engagement were implicitly established and explicitly enforced by participants. Often when participants were not engaged, they would address their peers directly, asking where they were, why they were not participating, and if they could propose a topic. A number of more active participants and the facilitator would often encourage others to join in a conversation or propose a new topic. Indeed, when participation was poor, participants and the facilitator would attempt to establish new rules for the group to renew engagement such as bringing up a new topic. Active facilitation led to more intimate displays of social support, earlier in the intervention. For instance, the facilitator in group three was not very active and topics remained superficial. Groups four and five had very active facilitators and began to speak about intimate issues very early in the intervention. Mentors in these groups were also willing to bring up issues related to their own lives in order to elicit empathy and encourage others to share. There was a sense of fear when the groups were closing, participants were keen to find ways of continuing the conversation.

### Social support and relationships

The largest single theme was social support (*n* = 2300 messages from sample, 80%). Messages under this theme related to checking in, giving support and group discussion. Checking in messages included daily greetings, farewells and ‘goodnights’. Such messages led into direct peer-to-peer social support including expressions of concern, affirmation or praise. These were often directed at individuals through the use of the message tagging function provided within Rocket.Chat®. Participants had an intimate memory of what their peers were going through and were careful to ask about such events. Such exchanges proved critical to building and maintaining the supportive space for participants.

**Table:** 

**Life (PM)**	@Leah and pavalu how are you today.
**Bronah**	am not fine feeling pain all over my body
**Mwansa**	@Bronah to bad dear get well soon and what is wrong?
**Life (PM)**	@Bronah @Broana could it be stress and since you just gave birth. Don’t be stressing too much with work. Getwell soon.

Participants also discussed their other sources of social support, including their relationships. 3% of messages were related to relationships (*n* = 79). Most participants were married, and hence topics within this theme related to emotions, wifely duties and marriage. Participants often discussed the importance of trust, love and sharing within their relationships with their husbands, and expressed gratitude for the positive elements of their relationships. They also referenced the need to maintain particular values and fulfil particular wifely duties in order to sustain their positive relationships. They discussed their ideal relationships as informed by religious principles, and their conjugal bond was divine and duty to be a good wife as a responsibility from God.

Participants also used the groups to seek advice when negative events occurred in their relationships; for instance, when their husband went missing, engaged in criminal activity or was having an affair. In the focus group discussion, one participant indicated, for example “[w]hen we had marital differences the group acted like a mediator. My husband and I would have differences but when you come to talk to people in the group you find that they will talk some sense into you, so the advice there helped to reduce conflict.”

Further, they discussed instances of domestic violence – physical, emotional or financial – that they experienced; and sought advice from their peers as to how to resolve these issues whilst maintaining a strong marriage. Advice from peers was often informed by patriarchal moral values, where participants encouraged each other to stay with their partners and not get angry in return. However, they also encouraged each other to be strong and resilient, whatever they may be going through.

**Table:** 

**Mumba**	[N]ow ladies whatever the situation your passing through don’t complain just pray to god at last bad things are going to become good things and we [learn] something in each and every challenge, that we can use to [encourage] other people.

Overall (Table 2), participants self-reported to perceive more (1.7% higher mean score) social support post-intervention, however, there is no evidence from the data to suggest that this was different from the way they felt pre-intervention [*Mean Difference = 1.7% (95%CI: − 10.8 - 14.1%), p-value = 0.4]*. Overall, participants self-reported a reduction in low self-esteem (0.3% reduction in the mean score) post-intervention, however, there is no evidence from the data to suggest that this was different from pre-intervention *[Mean Difference = − 0.3% (95%CI: − 9.0 - 8.3%), p-value = 0.9]*.

**Table 2:** Results of Paired t-test of the Pre-and Post-intervention Outcomes

	***Pre-Intervention Mean %***	***Post-Intervention Mean %***	p-value*
***Perceived Social Support***	23.3	25.0	0.8
***Low self esteem***	38.3	38.0	0.9
***HIV Knowledge***	73.6	74.9	0.7
***Internalized Stigma***	28.3	15.3	0.009
***Anticipated and Perceived Stigma***	48.7	52.7	0.4

Note: *Paired t-test

### HIV knowledge and ART adherence

Two percent of messages were related to the theme of Living with HIV (*n* = 68). Within this theme, the issues of medication, transmission and coping with status were referenced in conversation. With regard to medication, facilitators and peers often reminded participants to take their medication. Participants would often follow this up with reference to the benefits of taking medication to their health and the health of their child. Participants also discussed the barriers they had to accessing medication, resultant of poor health services and lack of funds.

**Table:** 

**Pavalu**	[Went] to the clinic they said that there is no medicine for the baby now they told us to buy now some people they don’t have money to buy now my question is when the baby don’t take medicine can the baby get affected
**Bronah**	@Pavalu am very happy for u the baby will be fine
**Bronah**	@Pavalu even me i was told that i need to buy

In the focus group discussion, participants indicated that it was very useful to have the group as a means of checking when they needed to go to the clinic. For instance, one participant stated “This was my first pregnancy, so for me some things looked normal, when I came to the group I asked for help, they told me to immediately go to the hospital, it was actually something serious.”

Guest speaker sessions were an important opportunity for participants to ask questions about issues relating to their medication including experience of side effects, the interaction of the medication with contraception, myths about the impact of the medication on their bodies and the impact of the medication on their baby.

**Table:** 

**Bamake_Confidence**	No it’s not true a pregnant woman who is HIV can give birth to a healthy baby
**Life (PM)**	Bamake that’s true ad if u also eating eating gud food ad u are taking ur medication very well.

Participants sought advice on alternative medications. Participants also discussed HIV transmission to their partners, peers and babies. Most messages related to transmission were questions, and participants had queries related to reinfection, breastfeeding, using protection, and how to care for their babies to prevent onward transmission.

**Table:** 

**Bronah**	@Dr. about contraceptive which one is good for the woman who is on medication
**Mwansa**	Doctor i heard people saying that this medicine we are taking it affect a brain is it true?
**Bronah**	When start medication what makes to be for gotten things
**Dr**	@Bronah The contraceptive depends on which one the woman prefers. The ARVs don’t affect the type of contraceptive to take. So when speaking to the person giving you the contraceptive you can mention to them the drugs you’re taking. There are very few which can affect. But it’s minor
**Pavalu**	Afternoon
**Dr**	@Mwansa Most ARVs do not affect the brain. They actually help because they are protecting it from infection. A few of them can have some slight side effects like dizziness and making you sleepy but it’s just a side effect, no damage
**Dr**	@Bronah It could be a side effect, which medication are you taking?

80% (24/30) of the participants self-reported to have been taking ARVs pre- and post-intervention; 25% (6/24) of these participants self-reported to have found it difficult taking ARVs pre-intervention compared with 28% (7/24) post-intervention. Less than 70% of the participants answered each of the HIV knowledge questions correctly both pre- and post-intervention, apart from the more technical ones on viral load, CD4 count and oral sex. Overall, participants seemed more knowledgeable on HIV post-intervention (13% higher score), however, we do not have enough evidence to suggest that their knowledge is different pre-intervention compared to pre-intervention *[Mean Difference = 12.8% (95%CI: − 5.7 to 82.8%), p-value = 0.7]*.

### Stigma

Two percent of messages were related to mental health and stigma (*n* = 56). Specifically, messages were related to positive or negative emotions, stress, depression and loneliness as well as the different manifestations of stigma – as experienced in their neighbourhood, as related to touch, as internalised as self-stigma and as related to shame for their condition. Such issues were almost always brought up first by the facilitator. Participants used the groups to share with their peers the experiences that impacted their mental health and sought advice from their peers as to what they should do. Their peers were able to provide both general emotional support and specific advice. Participants were also able to discuss instances where they were feeling lonely, distressed or socially isolated due to their status, and linked this to lack of support from their families or communities. Participants indicated that disclosure was a distressing issue within families. The group setting was important for some in accepting their status, for instance one participant indicated in the focus group “before Insaka my status (HIV) was like a burden but now I have started to love myself”. They used the group setting to refute myths they had heard in their communities.

**Table:** 

**Cathy**	What challenges do we face in the community and in our families due to our status
**Wamz**	Lack of support from our families ad in our community people start talk about u that u are HIV ad others also start laughing at u.

Participants also sought advice from their peers as to how to deal with anticipated stigma in daily life, when visiting the clinic or collecting medications. Peers were able to assuage such fears and encourage others to rise above the experience of stigma.

**Table:** 

**Bronah**	How do you feel when u ar gone to the clinic for correcting medication? And u see one of our neighbours or ur friends,do u feel ashamed,or what?
**Life (PM)**	@Bronah
**Life (PM)**	@Bronah Dear it feels good to see people you know doing the same thing you are doing and it makes you know that you are not alone in the same program.
**Life (PM)**	@Bronah @Leah and mwansa say something about what broana has asked.
**Bronah**	@Life What if they ar gone Ku under five.
**Life (PM)**	@Bronah dear I think all we need is just focus,minding our own business and if our neighbor happens to be a big mouth then 1 day they will leap their own harvest.
**Life (PM)**	So guy what do you say should one be running or hides when you see them

Overall (Table 2), participants self-reported to have experienced a reduction in internalized stigma post intervention (13% reduction in the mean score), and there is strong evidence from the data to show that this was different from the way they felt pre-intervention *[Mean difference = − 13.0% (95%CI: − 22.6% to − 3.5%), p-value = 0.009]*. Overall (Table 2), participants self-reported to anticipate and perceive more stigma post-intervention, however, there is no evidence from the data to suggest that this was lower pre-intervention *[Mean Difference = 4.0% (95%CI: − 6.1 to 14.1%), p-value = 0.4]*.

## Discussion

Evidence in this process evaluation of the Insaka intervention suggests that it is both feasible and acceptable for HIV positive adolescent pregnant women and new mothers. The feasibility of the intervention was facilitated by a number of factors. First, it was feasible to recruit from the antenatal clinics due to the intervention’s alignment with the existing standard of care that participants were receiving and their regular attendance. Interaction with the clinic was a key part of women’s lives as they had to pick up their ART medication and attend antenatal clinics before their delivery, attend the clinic for delivery and attend the under-five clinic after delivery. The identification of the clinic as a key touch point for recruiting this population is perhaps resultant from the successful scale up of clinic-based ANC and ART delivery services for this population in Zambia in order to prevent PMTCT [30]. This touch point can be further leveraged to deliver age-appropriate, accessible and convenient services mental health and psychosocial support services in Zambia and beyond [31, 32].

Second, the intervention was technically feasible because participants were able to use the platform, even if their literacy levels or English language skills were poor. The speech-to-text and spell check functions facilitated such interaction. Indeed, the mobile platform was engaging for 71% of participants. However, the times that participants engaged in conversations were different to those when they visited the clinic or might engage in face-to-face support groups. Women implied that their household responsibilities, caring for their husband and their children were key factors in this choice of time. This shows that the demands placed on women that might prevent them from attending a physical support group can be overcome through the use of a mobile phone-based platform. The intervention was sufficiently flexible, so women were able to access immediate and intimate psychosocial support from peers and professionals despite their complex lives. This observation resonates with recent advances in mobile-facilitated information and psychosocial support delivery to women of childbearing age in Southern African, including the SHM Foundation’s Project Kopano in South Africa [21]; and the MomConnect program run by the South African Ministry of Health [33]. Addressing the needs of this population through such flexible and accessible interventions is critical, as it acknowledges the ways in which women’s responsibilities for unpaid care work results in gender disparities in health seeking behaviour and access to health services [34].

The Insaka intervention was acceptable to this population for a number of reasons. First, the anonymity of participants made it easy for them to communicate and open up about the issues that were impacting their lives. The burden of stigma on people living with HIV is intensified in this population because of their youthfulness and the demands of motherhood [35]. As identified elsewhere in Southern Africa, women indicated that the gossip in their communities cause women to feel such stigma acutely, resulting in poor adherence to ART, poor mental health outcomes and an aversion to disclose their status [36]. The anonymity of the intervention was critical to overcoming this burden of stigma, such that women were able to build friendships, care for one another, and problem solve together without having to disclose their status. Women were able to bring up key issues that they were tackling in their homes, such as broken relationships, domestic violence and challenges they were facing with ART adherence in a safe and supportive space. This reveals the that the mobile phone platform can allow populations to overcome barriers of anonymity and confidentiality in community-based psychosocial support interventions [37].

Second, the peer-led structure of the social support groups was an important factor in acceptability as it encouraged a democratic and unintimidating space for women. The importance of peer-led social support interventions is well documented in psychosocial support provision for young people [38] and particularly for young mothers [39]. These findings contribute to the growing literature from Southern Africa that shows that the scale up of psychosocial support services can be facilitated through the use of virtual support groups for this population [40, 41]. Small acts of care were the foundation for group discussions, and it was only through the development of such a supportive environment that women were able to discuss challenging issues in their lives. As a result, the conversations were not limited to the imparting of knowledge from a central administrator. Participants were able to share strategies for dealing with the challenges they faced, such as reminding each other to take their medication or giving tips on how to manage difficulties in marriage. Participants indicated that the ability to share such insights allowed them to better care for their children, husband and families, as well as for themselves. The fact that the support groups were small, with a maximum of eight participants, meant that participants were able to get to know one another personally. Participants were able to follow the challenges that each other were facing and check in on participants who they knew were going through difficult times. Participants were keen to encourage those who were not engaged or who had not chatted for some time to return to the conversation. As a result, the support groups were intimate and allowed for the provision of tailored support. However, the objective of the intervention was normative – to support participants through situations of relational distress – rather than necessarily transformative – to change their behaviour or attitudes with respect to IPV or relationships. There is potential to leverage this space in a transformative way, especially to encourage participants to seek help when in situations of distress. This would require peer support workers to have a higher level of training and guidance in advocating for the needs of participants, crisis management and referral.

Third, the support groups provided participants with an important source of accurate information on pregnancy, child health and HIV. The content of the conversations as facilitated by peer mentors and guest speakers on HIV knowledge, medication and caring for a child, aligned with health information provided by healthcare professionals in antenatal clinics and provided a platform for participants to discuss their queries regarding such issues. As such, the platform provided an important place where women could bring their queries and dispel myths.

However, there were also a number of barriers to feasibility and acceptability. Twenty-nine percent of those recruited in the study could not participate due to relational, technological and literacy barriers. In our study, most women were educated to secondary level – a relatively high figure compared to the Zambian school net attendance ratio (NAR) for girls in secondary school was only 38% in 2018 [42]. In a country where there is a strong link between educational attainment and HIV testing [43], this means the Insaka intervention is acceptable and feasible for those with a basic level of literacy. However, those who did not participate also had a fairly high level of educational attainment. This alert us to a more complex picture around phone use behaviour, and to imperative of understanding the needs of this population around the mobile phone, and to provide specific training and support for this population when engaging them in mobile phone-based interventions. Further, face-to-face services must be designed to complement digital provisioning such that those who are unable to participate in such interventions are still well served. This population is also at high risk of Intimate Partner Violence (IPV). In Zambia, 43% of women aged 15–49 years report lifetime experience of physical and/or sexual IPV and 27% of women reported physical and/or sexual IPV in the past 12 months [44]. The introduction of the mobile phone into such a high-risk environment could lead to jealousy or suspicion, changing the power dynamics in the household. Hence, it is critical that a team of trained professionals are able to engage partners or other family members in the consent process in order to safeguard participants.

### Limitations of the study

The study was insufficiently powered to detect real differences if they exist, and therefore, there is need to increase the sample size to make reliable conclusions. Implementation of this intervention with a larger sample size is necessary to understand its impact on biological outcomes, such as viral suppression and mother-to-child transmission; and on measurable mental health outcomes, such as depression and anxiety, internalised stigma and hope. The focus group discussion did not include any participants who did not participate or dropped out of the study, potentially introducing bias to the feasibility and acceptability data. In future iterations of the model, they should be followed up and their reasons for dropping out should be understood more deeply. The literacy and technological literacy barriers indicate that participation in the study was limited to those with secondary educational attainment. In future iterations of the model, it will be important to better understand and meet the literacy and technological needs of participants so as to expand the reach of the intervention.

## Conclusion

Findings from this study suggest that mobile phone support groups are an acceptable and feasible means of delivering psychosocial support to adolescent pregnant women living with HIV in Zambia. The peer-led, small group structure of the intervention proved important for the provision of support, while guest speaker sessions proved effective at delivering accurate information. However, some participants experienced challenges participating in the intervention due to poor literacy, technological literacy, or relational issues with partners. Further research could be done on how the intervention could be integrated into the health system and allow women to overcome barriers of stigma to access care and complement the provision of face-to-face services. Insights from the intervention could be shared with health care professionals to orient behaviour toward the needs of this population in order to augment the quality of care they receive.

## Supplementary Information

**Additional file 1.** Project Insaka questionnaire.

**Additional file 2.** Insaka Focus Group Discussion Guide

## Data Availability

Anonymised text message data used for this publication; and questionnaire data is available on request of the authors.

## Abbreviations

ART: Anti-Retroviral Treatment
HIV: Human Immunodeficiency Virus
IPV: Intimate Partner Violence
MCH: Mother and Child Health
PM: Peer Mentor
