# Correction to: Insaka: mobile phone support groups for adolescent pregnant women living with HIV

**DOI:** 10.1186/s12884-021-04232-3

**Published:** 2021-12-02

**Authors:** Nikita Simpson, Anna Kydd, Mwelwa Phiri, Madalitso Mbewe, Lucheka Sigande, Thomas Gachie, Malebo Ngobeni, Tebogo Monese, Zuzana Figerova, Hugo Schlesinger, Virginia Bond, Steve Belemu, Musonda Simwinga, Ab Schaap, Maurice Biriotti, Sarah Fidler, Helen Ayles

**Affiliations:** 1 Department of Anthropology, London School of Economics and Political Science, London, UK; 2 SHM Foundation, 20-22 Bedford Row, London, UK; 3 Zambart, Lusaka, Zambia; 4 Department of Global Health and Development, London School of Hygiene and Tropical Medicine, London, UK; 5 Department of Infectious Diseases, Imperial College, London, UK; 6 Clinical Research Department, London School of Hygiene and Tropical Medicine, London, UK

**Correction to: BMC Pregnancy Childbirth 21, 663 (2021)**

**https://doi.org/10.1186/s12884-021-04140-6**

Following publication of the original article [1], the authors identified an error in the affiliations of Nikita Simpson.

Nikita Simpson is only affiliated to “1 Department of Anthropology, London School of Economics and Political Science, London, UK.” and “2 SHM Foundation, 20-22 Bedford Row, London, UK.”

The original article [1] has been corrected.
