# Supplementary material for: Insaka: mobile phone support groups for adolescent pregnant women living with HIV
Source: BMC Pregnancy Childbirth. 2021 Sep 30;21:663. doi: 10.1186/s12884-021-04140-6 (PMC8482634; doi:10.1186/s12884-021-04140-6)
Supplement: Supplementary file 1 — Additional file 1. Project Insaka questionnaire. [file 12884_2021_4140_MOESM1_ESM.docx]

**APPENDIX D: PROJECT INSAKA QUESTIONNAIRE – 2018**

| **Thematic Area** | **Question** | **Type of answer** |
| --- | --- | --- |
| **Personal Details** | 1. Participant ID: [Automatic] | UIN |
|  | 2. What would you like us to call you in the group? You can choose any ‘nickname’ you like: | Text: Open for participant to fill in |
|  | 3. Are you married/unmarried: | Married/unmarried |
|  | 4. Date of birth | dd/mm/yyyy |
|  | 5. Age: | Number |
|  | 6. Are you in school/tertiary/employed at the moment: (please tell us which) | Choices |
|  | 7. What clinic do you attend? | Open to fill in |
|  | 8. What city/township/village do you live in? | Open |
|  | 7. What is the highest grade/ level of education you have achieved? | Open for participant to fill in |
|  | 8. Have you been pregnant before? If yes, how many children have you had before? | Yes/No |
| Adapted from Rammstedt, B. & John, O. P. (2007). Measuring personality in one minute or less: A 10 item short version of the Big Five Inventory in English and German. Journal of Research in Personality, 41, 203‐212. BFI-10 | I see myself as someone who ... | Strongly Disagree, Disagree, Neutral, Agree, Strongly agree |
|  | 1. ... is reserved |  |
|  | 2. ... is generally trusting |  |
|  | 3. ... tends to be lazy |  |
|  | 4. ... is relaxed, handles stress well |  |
|  | 5. ... has few artistic interests |  |
|  | 6. ... is outgoing, sociable |  |
|  | 7. ... tends to find fault with others |  |
|  | 8. ... does a thorough job |  |
|  | 9. ... gets nervous easily |  |
|  | 10. ... has an active imagination |  |
|  |  |  |
| **Living circumstances** | 1. Who looks after you?  (please tell us what relationship this person is to you - mother/ brother/sister/cousin/friend/teacher/aunt etc.) | Open to fill in |
| Mother | 2. Is your mother still alive? (a, b,c) | Yes, No, I don't know |
|  | a. Yes: |  |
|  | Does she live in the same house as you? | Yes/No |
|  | If she doesn’t live with you, how often do you see her? | Open to fill in |
|  | b. No: |  |
|  | What was the cause of her death? | Open to fill in |
|  | c. I don’t know | Move to next |
| Father | 3. Is your father still alive? | Yes, No, I don't know |
|  | a. Yes |  |
|  | Does he live in the same house as you? | Yes/No |
|  | If he doesn’t live with you, how often do you see him? | Open to fill in |
|  | b. No |  |
|  | What was the cause of his death? | Open to fill in |
|  | c. I don’t know |  |
| Home circumstances | 1. Does your family ever struggle with money? | Yes/No |
|  | 2. Is there ever a time when there is not enough food in your home? |  |
|  | 3. Do you have running water in your home? |  |
|  | 4. Do you have electricity in your home? |  |
|  | 5. Do you have to look after anybody else at home? |  |
|  | a. If yes, who do you look after? (Please tell us what relationship this person is to you - mother/ brother/sister/cousin/friend/teacher/aunt etc.) | Open |
| **HIV diagnosis** | 1. When did you find out your HIV status? | Open |
|  | 2. Who told you about your status? | Open |
|  | 3. How old were you when you were diagnosed? | Number |
| **HIV Knowledge Quiz** | 1. ARVs can cure people of HIV and AIDS | Yes/No/Don't know |
|  | 2. A person with HIV always looks very thin or unhealthy in some way |  |
|  | 3. Coughing and sneezing spread HIV |  |
|  | 4. A person can get HIV by sharing a glass of water with someone who has HIV |  |
|  | 5. Showering or washing one’s genitals/private parts, after sex keeps a person from getting HIV |  |
|  | 6. ARVs are medicines that help HIV positive people to live healthier lives |  |
|  | 7. HIV and AIDS are the same thing |  |
|  | 8. A low viral load means that someone's immune system is weak |  |
|  | 9. A person can get HIV from oral sex |  |
|  | 10. There is a way for someone who has HIV to get pregnant without passing the virus on to her child |  |
|  | 11. Having a low viral load guarantees that you won’t transmit HIV to someone else |  |
|  | 12. People with HIV can’t live healthy productive lives |  |
|  | 13. A low CD4 count means someone's immune system is strong |  |
| **Internalised Stigma** | Getting HIV is a punishment for bad behaviour | Yes/No; Agree/Disagree |
|  | I think less of myself because I have HIV |  |
|  | People are right to be afraid of me because I have HIV |  |
|  | I feel that it is my fault that I got HIV |  |
|  | I must have done something wrong to deserve getting HIV |  |
|  | I feel ashamed that I have HIV |  |
|  | When people know I have HIV I feel uncomfortable around them |  |
|  | If I was in public or private transport and someone knew I had HIV they would not sit next to me |  |
|  | My neighbours would not like me living next door if they knew I had HIV |  |
|  | I would understand if people rejected my friendship because I am HIV positive |  |
|  | Most employers would not employ me because I am HIV positive |  |
|  | If I drank from a tap and people knew I had HIV they would not drink from the same tap |  |
| **Berger stigma scale selected Q's including anticipated and perceive stigma** | 1. In many areas of my life, no one knows that I have HIV | SD, D, A, SA |
|  | 2. I feel guilty because I have HIV |  |
|  | 3. People's attitudes about HIV make me feel worse about myself |  |
|  | 4. Having HIV makes me feel dirty |  |
|  | 5. Telling someone I have HIV is risky |  |
|  | 6. I work hard to keep my HIV a secret |  |
|  | 7. It is easier to avoid new friendships than worry about telling someone that I have HIV |  |
|  | 8.  Most people think that a person with HIV is disgusting |  |
|  | 9. Most people with HIV are rejected when others find out |  |
|  | 10.  Since learning I have HIV, I feel lonely |  |
| **PLWHIV Stigma Index** | 1. Since finding out your status have you been fearful of any of the following things happening to you – whether or not they actually have happened to you? |  |
| Anticipated stigma | a. Being gossiped about | Yes/No |
|  | b. Being verbally insulted, harassed and/or threatened |  |
|  | c. Being physically harassed and/or threatened |  |
|  | d. Being physically assaulted (violence) |  |
| Experienced stigma | 2. How often have you been aware of being gossiped about because of your HIV status? | Most, Some, Never |
|  | 3. In the last 12 months, how often have you been excluded from social gatherings or activities because of your status? | Most, Some, Never |
| Resilience | 4. Have you ever confronted, challenged or educated someone who saying bad things about People living with HIV? | Yes/No |
|  | 5. Have you confronted, challenged or educated someone who was stigmatising and/or discriminating against you? | Yes/No |
|  | 6. Have you ever done anything to support other people living with HIV? | Yes/No |
| Perceived Social Support | 1. There is a special person who is around when I am in need. | SD, D, N, A, SA (1-5) |
|  | 2. There is a special person with whom I can share my joys and sorrows. |  |
|  | 3. My family really tries to help me. |  |
|  | 4. I get the emotional help and support I need from my family. |  |
|  | 5. I have a special person who is a real source of comfort to me. |  |
|  | 6. My friends really try to help me. |  |
|  | 7. I can count on my friends when things go wrong. |  |
|  | 8. I can talk about my problems with my family. |  |
|  | 9. I have friends with whom I can share my joys and sorrows. |  |
|  | 10. There is a special person in my life who cares about my feelings. |  |
|  | 11. My family is willing to help me make decisions. |  |
|  | 12. I can talk about my problems with my friends. |  |
| General Social Support | Do you have someone to turn to for suggestions about how to deal with a personal problem? | Yes/No |
|  | Do you have someone to help with daily chores if you were sick? |  |
|  | Do you have someone to show you love and affection? |  |
|  | Do you have someone to do something enjoyable with? |  |
|  | Do you have someone who you can ask when you need money, clothes, books? |  |
| Rosenberg Self-esteem scale | 1. On the whole, I am satisfied with myself | SA, A, D, SD (1-4) |
|  | 2. At times I think I am no good at all. |  |
|  | 3. I feel that I have a number of good qualities. |  |
|  | 4. I am able to do things as well as most other people. |  |
|  | 5. I feel I do not have much to be proud of. |  |
|  | 6. I certainly feel useless at times. |  |
|  | 7. I feel that I'm a person of worth, at least on an equal plane with others. |  |
|  | 8. I wish I could have more respect for myself. |  |
|  | 9. All in all, I am inclined to feel that I am a failure. |  |
|  | 10. I take a positive attitude toward myself. |  |
| Disclosure | 1. Are you able to speak to the adults in your life about things that are important to you? (Yes/No) | Yes/No |
|  | a. Would you be able to talk to the adults in your life about sex? |  |
|  | b. Have you ever spoken to the adults in your life about sex? |  |
|  | c. Would you be able to talk to your parents/ adults in your life about HIV? |  |
|  | d. Have you ever spoken to your parents/adults in your life about HIV? |  |
|  | 2. Who besides your doctor knows about your HIV status? (please tell us what relationship this person is to you - mother/ brother/sister/cousin/friend/teacher/aunt etc.) | Open |
|  | 3. Who have you told about your HIV status? (please tell us what relationship this person is to you - mother/ brother/sister/cousin/friend/teacher/aunt etc.) |  |
|  | 4. Who would you like to tell about your HIV status but haven’t yet? (please tell us what relationship this person is to you - mother/ brother/sister/cousin/friend/teacher/aunt etc.) |  |
|  | 5. Would you be comfortable talking to your friends about HIV? | Yes/No |
|  | 6. Would you be able to share your HIV status with your friends? |  |
| Disclosure: Relationships | 1. Do you currently have a girlfriend/boyfriend? | Yes/No |
|  | 2. Have you spoken/Would you speak to your boyfriend/girlfriend about HIV? |  |
|  | 3. Would you tell/ have you told your partner your HIV status? |  |
|  | 4. Do you intend to tell him/her? | (Only if previous answer is no & in a relationship) (Yes/No) |
| Self-reported adherence | 1. Are you taking ARVs?  b. if No why? (open) | Yes/No  (If yes, skip to Q2, In No to Q1b) |
|  | 2. When did you start taking ART/ How long have you been taking ART? | Open |
|  | 3. Do you ever find it difficult to take your treatment? | Yes/No |
|  | 4.  Everyone struggles to take medication as prescribed from time to time, what are some of the reasons you have struggled to take your meds? | Open |
|  | 5. How many pills has the doctor told you to take a day? | Number |
|  | 6.  How often do you miss taking your medication? | Select the answer that best describes your medication habits |
|  | a. Once every six months |  |
|  | b. Once a month |  |
|  | c. Twice a month |  |
|  | d. Once a day |  |
|  | e. Once a week |  |
|  | f. Twice a week |  |
|  | 8. How many appointments have you missed in the last year? | Number |
|  | 8. Do you know the name of the medication that you are taking? | Yes/No |
|  | 9. Do you feel that you understand how to take the medication well? | Yes/No |
| Relationship with healthcare practitioner | 9. Do you talk to your healthcare practitioner/doctor about your medications? | Yes/No |
|  | 10. Do you talk to your healthcare provider if you are having problems with your medication? |  |
|  | 11. Do you feel able to tell your doctor if you have missed taking your medication? |  |
|  | 12. Do you talk to your doctor about health problems other than HIV? |  |
|  | 13. Do you talk to your doctor about your feelings? |  |
| Healthcare Practitioner reported adherence | a.     Viral load/ control |  |
|  | b.     CD4 count |  |
|  | c.     Medication collection |  |
|  | d.     Appointments attended |  |
| Breastfeeding(post-partum) | 1. Has your baby ever been given anything other than breast milk, since it was born? (if answer is no proceed to question 1 C.) | Yes []  No []  Don’t know [] |
|  | 1. If yes, what was given (tick all that apply) | Infant formula []  Water []  Sugar water []  Other fluids please do tell us what |
|  | 1. Why was your baby given supplement | I requested it (ask why)[]  Dr or other medical staff recommended it but didn’t say why []  Dr or other medical staff recommended it because (give reason)…….. |
|  | 1. How are you feeding your baby | Breastfeeding exclusively []  Breast feeding + milk substitutes []  Breast milk substitutes(no breast feeding at all[]please describe ….. |
|  |  |  |
|  |  |  |
| Breast feeding knowledge and information (for both pregnant and post-partum) | 1. Have you received any advice on breastfeeding (if answer is no, proceed to 2d.) | Yes []  No [] |
|  | 1. If yes, what kind of breast feeding advice have you received? | How long to breast feed? []  How long not to breast feed []  When to give baby food []  When not to give baby food  Other specify… |
|  | 1. Please tell us who you got this information from | Probe for  Medical staff (Dr, Nurse)  Lay health workers  Friends, Family members, random person from within or outside the community either pregnant or not, male or female) |
|  | 1. Was this information useful… | Yes []  No [] |
|  | Please explain why for answer in C above | Open (probe for application of information recived) |
|  | 1. What kind of information about breast feeding would you like to receive? | Open |
|  | 1. What kind of myths have you heard about women LWH and breast feeding? | Open |
