# Supplementary material for: Insaka: mobile phone support groups for adolescent pregnant women living with HIV
Source: BMC Pregnancy Childbirth. 2021 Sep 30;21:663. doi: 10.1186/s12884-021-04140-6 (PMC8482634; doi:10.1186/s12884-021-04140-6)
Supplement: Supplementary file 2 — Additional file 2. Insaka Focus Group Discussion Guide [file 12884_2021_4140_MOESM2_ESM.docx]

**Insaka Focus Group Discussion Guide**

Before starting the FGD, the facilitator reminds the group of:

- The purpose of the FGD
- Ground rules for FGD, including importance of confidentially and use of

pseudonyms

- That notes will be taken from the session.

1. What did you enjoy the most about project Insaka?
2. What did you enjoy the least about this project?
3. What were the advantages of this project?
4. What did you find the disadvantages of the project were?
5. How did taking part in this project impact your life?
6. What were your experiences of using the phones and Rocket chat?
7. Did you have any problems expressing yourself on the group?
8. Did you have any other comments about the intervention?
